# The Effects of Estrogen Receptors' Antagonist on Brain Edema, Intracranial Pressure and Neurological Outcomes after Traumatic Brain Injury in Rat

**DOI:** 10.7508/ibj.2015.03.006

**Published:** 2015-07

**Authors:** Fatemeh Dehghan, Mohammad Khaksari, Elham Abbasloo, Nader Shahrokhi

**Affiliations:** 1 *Physiology Research Center, Institute of Neuropharmacology and Physiology Department, Kerman University of Medical Sciences, Kerman, Iran;*; 2 *Neuroscience Research Center, Institute of Nneuropharmacology and Physiology Department, Kerman University of Medical Sciences, Kerman,* Iran

**Keywords:** Estrogens, Intracranial pressure (ICP), Brain edema

## Abstract

**Background:**

In previous studies, the neuroprotective effect of 17β-estradiol in diffuse traumatic brain injury has been shown. This study used ICI 182,780, a non-selective estrogen receptor antagonist, to test the hypothesis that the neuroprotective effect of 17β-estradiol in traumatic brain injury is mediated by the estrogen receptors.

**Methods:**

The ovariectomized rats were divided into eight groups. Brain injury was induced by Marmarou’s method. Estrogen was injected 30 minutes after traumatic brain injury, and ICI 182,780 was injected before traumatic brain injury and also before estrogen treatment. In one group only ICI 182,780 was injected. The brain water content and Evans blue dye content were measured 24 and 5 hours after traumatic brain injury, respectively. The neurologic outcomes and intracranial pressure were assessed before, 4, and 24 hours after traumatic brain injury.

**Results:**

Brain water content and Evans blue content were less in estrogen-treated group comparison to vehicle group. ICI 182,780 eliminated the effects of estrogen on brain edema and brain blood barrier permeability. Intracranial pressure was increased significantly after trauma, and estrogen decreased intracranial pressure at 4 and 24 hours after traumatic brain injury in comparison to vehicle. This inhibitory effect was also eliminated by treatment with ICI182,780. ICI 182,780 also inhibited the estrogen induced increase in neurologic outcomes following traumatic brain injury. However, the use of ICI 182,780 alone had no neuroprotective effect after traumatic brain injury.

**Conclusion:**

The results suggest that classical estrogen receptors have probably a role in the neuroprotective function of estrogen following traumatic brain injury.

## INTRODUCTION

Traumatic brain injury (TBI) is the main cause of disability and mortality in young adults such that its annual occurrence in the United States is 200 in 100,000 [1] individuals. It has been revealed that TBI causes damage to the brain blood barrier (BBB) and increase in its permeability and consequently increase in brain edema and intracranial pressure (ICP) [2]. Also, it has been reported that mortality in TBI is induced by brain edema and inflammatory responses. Up to now, an effective pharmacological treatment remains to be found [3].

It has been accepted that estrogen has a strong neuroprotection effect on different brain injury models [4]. Using female sexual hormones after TBI show some protective effects on the injured brain , such as protection of BBB, reduction of post-injury oxidative stress, and edema [5]. In addition, it has been reported that estrogen causes protection against brain injury and neurodegenerative diseases; prescription of estrogen decreases brain injury after focal ischemia [6]. Our previous study has shown that estrogen is able to reduce brain edema, protect BBB and reduce ICP [7] following TBI. Also in our recent study, we found that brain edema and ICP are low in TBI in the stage of the estrous cycle when estrogen level is high [7].

17β-estradiol (E_2_) is the most potent source of endogenous estrogen. Many E_2_functions are carried out through classic receptors, including estrogen receptor-α (ERα) and estrogen receptor-β (ERβ). These receptors have roles in both classic and non-classic signaling pathways. For example, the mediation of both receptors in the neuroprotective effect of estrogen [8], the expression of neuroinflammatory genes in the frontal cortex through ERα and ERβ [9] and the reduction of neuroprotective effect of estrogen in ischemia after the use of both receptor antagonists [10]

ICI 182,780 is an estrogenic compound known as a pure antagonist to estrogen that has an affinity similar to estrogen to its receptors in the brain [11]. This compound has also a similar affinity to both ERα and ERβ receptors. ICI 182,780 functions by signalling estrogen receptors to oppose through preventing dimerization of the receptor [12], the disturbance in nuclear receptor sites, and destroying the receptor [13]. The usage of ICI 182,780 alone exacerbates the effects of brain ischemia [14]. In spite of this, it has been reported that this compound may modify the expression of the gene independent of estrogen, or this compound may also have relative agonistic effects [15].

In this study, the ICI 182,780 compound was used along with estrogen to evaluate the possible role of ERα and ERβ in the neuroprotective effects of 17β-estradiol, including the reduction of the brain edema, restoration of BBB, reduction of the ICP, and improvement of the neurologic scores in diffuse TBI. Another objective of this study was to test the possible agonistic effects of ICI 182,780 on this model of the brain damage.

## MATERIALS AND METHODS

***Experimental procedures:***

***Animals. ***This study was conducted in accordance with the guidelines for the animal experiments of Kerman University of Medical Sciences. The protocol was approved by the ethics committee (No: k/92/314) of the university in accordance with the internationally accepted principles for laboratory animal use and care, as found in the European Community Guidelines (EEC Directive of 1986; 86/609/EEC) or US guidelines (NIH publication #85–23, revised in 1985). Animals (adult female Albino N-Mary rats, weighing 200-250 g) were housed in an air-conditioned room at 22–25ºC with a 12 h light:12 h dark cycle and free access to food and water.

***Bilateral ovariectomy. ***The animals were first anesthetized by the injection of 60 mg/kg thiopental (i.p.). The subabdominal area of the body was then shaved, and an incision of 2 cm was made. The skin, fascia, and abdominal muscles were opened, and fats and intestines were sheared off until the uterus and its tubes were exposed. Catgut 4 thread was then twisted around the tube of uterus and vascular base of the ovaries in the proximal area and cut from the distal area. Then 1-2 ml saline solution into the abdomen and the muscles, and skin was replaced. The incision was stitched using 0-2 silk thread, and the wound was washed with betadine solution. In addition, all experimental animals were ovariectomized (OVX) two weeks before the experiments to avoid interference due to the estrus cycle [16].

***Experimental protocols. ***The animals were randomly divided into eight groups (n = 7 in each group) as follows (all drugs were injected i.p.): (i) Sham group: OVX rats underwent preparation procedure of brain trauma but were not exposed to brain trauma; (ii) TBI group: OVX rats exposed to brain trauma and received no medicine; (iii) TBI + OIL group: OVX rats received an injection of an equal volume of vehicle (sesame oil, which were used as estrogen solvent) 30 minutes after TBI [5]; (iv) TBI + E_2_ group: OVX rats received an injection of estrogen (33.3 μg/kg) 30 minute after TBI [17]; (v) TBI + ICI + E_2 _group: OVX rats received an injection of ICI 182,780 (4.0 mg/kg) two times, 24 hours apart before TBI and then received an injection of estrogen (33.3 μg/kg) 30 minute after TBI; (vi) TBI + DMSO + E_2_ group: OVX rats received an injection of an equal volume of vehicle (DMSO, which was used as ICI182,780 solvent) two times, 24 hours apart before TBI, and then received an injection of estrogen (33.3 μg/kg) 30 minute after TBI; (vii) TBI + ICI group: OVX rats received an injection of ICI 182,780 (4.0 mg/kg) 30 minute after TBI; (viii) TBI + DMSO group: OVX rats received an injection of an equal volume of vehicle (DMSO) 30 minute after TBI.

***Drugs. ***17-β-estradiol and sesame oil were obtained from Aburaihan Pharmaceutical (Tehran, Iran), and ICI 182,780 was purchased from Sigma (China).

***Model of diffuse TBI. ***All animals were incubated before TBI. The TBI method of the diffuse type was induced by the Marmarou’s method [18] using a TBI induction device made by the Dept. of Physiology, Kerman University of Medical Sciences (Kerman, Iran). The protocol was as follows: a 300-g weight was dropped from a 2-m height on the head of the anesthetized [gas-mixture of isoflurane/N_2_O/O_2_ (2%, 66%, 32%)] female rats while a metal disc (stainless steel, 10 mm in diameter, 3 mm thick) was attached to the animal’s skull. After the induction of the trauma, the rats were immediately connected to a respiratory pump (TSA animal respiratory compact, Germany). After spontaneous breathing was restored, the intratracheal tube was removed, and following recovery the rats were placed in individual cages.

***Determination of brain edema. ***The brain edema of each animal was assessed by measuring brain water content. The anesthetized animals were sacrificed 24 h after TBI, the brain was removed, and brain samples were placed in pre-weighed glass vials and weighed (wet weight). The lids were removed, and the vials were placed in an incubator (Memmert, Germany) at 60ºC for 72 h, and then reweighed (dry weight). The percentage of water in each sample was then calculated using a formula published previously [17]:

Brain water content (%) = [(wet weight - dry weight)/wet weight] × 100

***Determination of BBB permeability. ***The degree of BBB permeability was assessed by measuring Evans blue dye leakage with a slight modification [17]. Four hours after TBI, a dose of 20 ml/kg Evans blue dye was injected intravenously through tail vein. The animals were anesthetized 5 h after TBI and perused using saline to remove intravascular Evans blue dye. The brains were removed, then dissected, weighed, and stored at –80 8C for quantitative measurement. Brain samples were homogenized in 1 mL of 0.1 mol/L PBS, and 0.7 mL of 100% (w/v) trichloroacetic acid was added to it, and centrifuged. After centrifugation for 30 min at 1,000 ×g, the absorbance of Evans blue in supernatant was measured at 610 nm using a spectrophotometer (UV/VIS, Spectrometer, UK). The amount of extravasated Evans blue dye content was quantified as μg/g brain tissue.

***ICP evaluation. ***The ICP was determined by using ICP monitoring system made by MobinKahroba Kimia Co. (Iran) and Dept. of Physiology, Kerman University of Medical Sciences. The anesthetized animal (with 68% N_2_O + 30% O_2_ + 2% halothane) was placed in stereotaxic instrument in the way that its shead was placed in midsagittal plane, and the anterior-posterior point was located at about midpoint between the occipital crest and the lambda suture. After indenting cistern magna area, a no. 20 needle connected to an E50 tube of ICP monitoring device was entered into the cisterna magna area and transferred the pressure to the transducer. By a recording system (AD Instruments, Australia), the pressure was recorded before the trauma induction and 4 and 24 h after TBI [5].

***Evaluation of neurologic outcomes. ***Based on veterinary coma scale (VCS), the neurologic outcome was scored in the range of 3-15 that was the sum of three parts: motor function (score range 1-8), eye function (score range 1-4), and respiration (score range 1-3). According to VCS criteria, higher scores indicated better neurological outcomes, and lower scores indicated worse neurological outcomes. In the present study, the outcomes were measured one hour before trauma induction and immediately after trauma (time 0), and measurements were continued 4 and 24 hours post TBI [5].

***Statistics. ***The normality of data was checked using Shapiro-Wilk’s W test. A mixed design analysis of variance was used to evaluate the interaction between the times of ICP and VCS measurement and the groups (*P* < 0.001, corrected with Greenhouse-Geisser); therefore, the data was analyzed at different times by one-way analysis of variance (ANOVA). Data of water content and Evans blue content were analyzed using one way ANOVA. In addition, LSD test was used for post hoc analysis. Quantitative data were presented as mean ± SEM, and the level of significance was considered at *P*<0.05.

## RESULTS

**Fig. 1:**
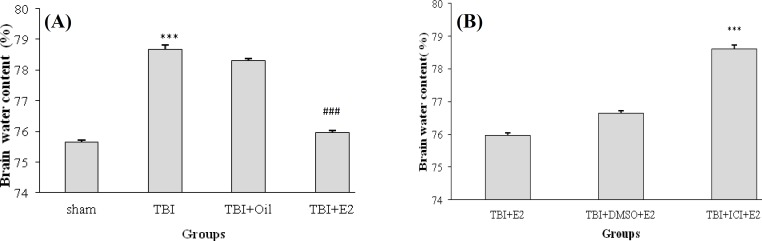
**. **The brain water content (%) following traumatic brain injury in rats (n = 7/group). Data are represented as mean ± SEM. ^***^P < 0.001;. TBI vs sham. ^###^*P* < 0.001; TBI + E_2_vs TBI and TBI + OIL (A). ^***^P < 0.001; TBI + ICI + E_2_ vs TBI + E_2_, and TBI + DMSO + E_2_ (B). TBI, traumatic brain injury; E_2_, estradiol; ICI, ICI 182,780.

***Brain edema. ***The water content of the brain in different groups at 24 h post TBI is shown in Figure 1. Figure 1A shows that the brain water content in TBI group is significantly higher than in sham group (*P* < 0.001). On the other hand, the brain water content in TBI + E_2_ was signiﬁcantly less than that in TBI and TBI + OIL groups (*P* < 0.001), whereas there was no significant difference between TBI and TBI + OIL groups. Figure 1B shows that brain water content in TBI + ICI + E_2_ group is more than in TBI + E_2_ (*P* < 0.001) and TBI + DMSO + E_2 _(*P* < 0.001) groups. The brain water content between TBI + ICI and TBI + DMSO groups was not significant (data not shown).

**Fig. 2.**
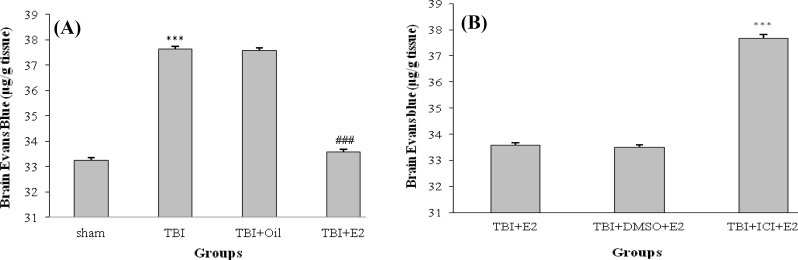
The Evans blue dye content (µg/g tissue) following traumatic brain injury (TBI) in rats (n = 7/ group). Data are represented as mean ± SEM.***P < 0.001; TBI vs. sham. ### *P* < 0.001; TBI + E_2_ vs TBI and TBI + OIL (A). ***P < 0.001; TB + ICI + E_2_ vs TBI + E_2 _and TBI + DMSO + E_2_ (B). E_2 _(estradiol); ICI (ICI 182,780)

***BBB permeability*** *. *The Evans blue dye content in all groups at 24 h after TBI is shown in Figure 2. Figure 2A shows that the Evans blue dye content in TBI group is significantly more than in sham group (*P* < 0.001). TBI + E2 showed a signiﬁcant decrease in the Evans blue content compared with TBI and TBI + OIL groups (*P* < 0.001), whereas no difference in the level of Evans blue content was observed between TBI and TBI + OIL groups. Figure 2B shows that Evans blue content in TBI + ICI + E2 group is more than in TBI + E2 (*P* < 0.001) and TBI + DMSO + E2 (*P* < 0.001) groups. The level of Evans blue content between TBI + ICI and TBI + DMSO groups was not significant (data not shown).

**Fig. 3.**
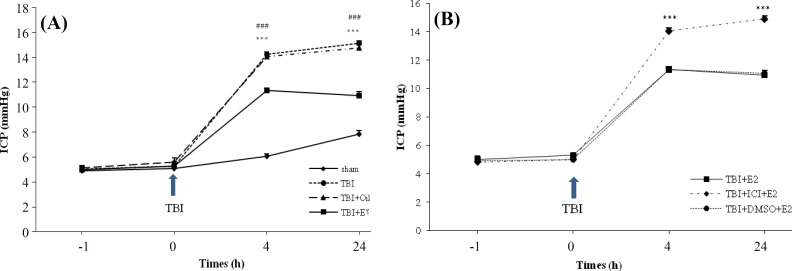
The effect of traumatic brain injury (TBI) on intracranial pressure (ICP) in different groups (n = 7 in each group). The data are represented as mean ± SEM. ***P < 0.001; at 4 and 24 h after TBI for TBI vs. sham, ### *P* < 0.001; at 4 and 24 h after TBI for TBI + E_2_ vs TBI and TBI + OIL groups (A). ***P < 0.001; at 4 and 24 h after TBI for TBI + ICI + E_2_ vs TBI + E_2 _and TBI + DMSO + E_2_ (B). E_2_ (estradiol), ICI (ICI 182,780).

***ICP measurements.*** Figure 3 illustrates the ICP alterations in traumatic groups in all studied groups at different times after TBI. Figure 3A shows that before TBI, there was no significant difference among groups regarding to ICP. The induction of trauma caused an increase in ICP so that 4 and 24 hours after trauma, the ICP was increased in all traumatic groups compared to sham group.

However, there was a significant difference in ICP in TBI + E_2 _compared to TBI and TBI + OIL groups (*P* < 0.001), whereas there was no significant difference in ICP between TBI and TBI + OIL groups. Figure 3B shows the ICP changes in different groups 4 and 24 h post TBI. There was a significant difference between TBI + ICI + E_2_ with TBI + E_2_ and TBI + DMSO + E_2_ (*P* < 0.001). There was no significant difference in ICP between the TBI + ICI and TBI + DMSO groups (data not shown). 

**Fig. 4:**
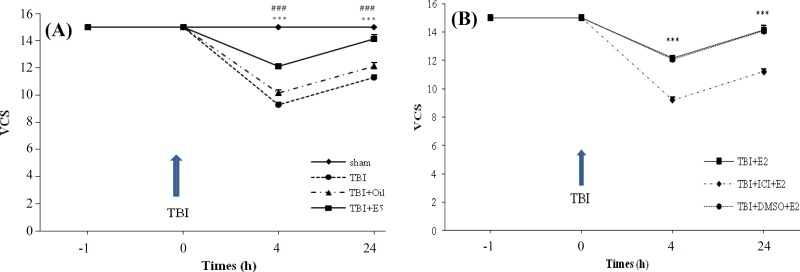
The effect of traumatic brain injury (TBI) on Veterinary coma scale (VCS) in different groups (n = 7 in each group). The data are represented as mean±SEM. *** *P* < 0.001; at 4 and 24 h after TBI for TBI vs sham ,### *P *< 0.001; at 4 and 24 h after TBI for TBI + E_2_vs TBI and TBI + OIL groups (A).^***^ *P* < 0.001; at 4 and 24 h after TBI for TBI + ICI + E_2_vs TBI + E_2_ and TBI + DMSO + E_2_(B). TBI, traumatic brain injury; E_2_, estradiol; ICI, ICI 182,780

***Evaluation of neurological outcomes. ***Changes in the neurological scores (VCS) of the sham and differently treated OVX rats at different times after TBI are shown in Figure 4. Figure 4A shows a marked decrease in VCS score in the TBI group in comparison with the sham groups immediately after TBI. This damage was not significantly affected by the administration of the oil solution. A decrease in VCS score in the TBI and TBI + OIL groups were significantly inhibited by treatment with E2 (*P* < 0.001). Figure 4B shows the VCS score changes in different groups. Four and 24 hours following TBI, there was a significant difference between TBI + ICI + E_2_ with TBI + E_2_ and TBI + DMSO + E_2_ (*P* < 0.001). In addition, there was no significant difference in the VCS score between the TBI + ICI and TBI + DMSO groups (data not shown).

## DISCUSSION

In the present study, the ICI 182,780 compound was used along with estrogen to ascertain the role of classical estrogen receptors in the neuroprotective effects of estrogen, and three major findings were acquired. First, estrogen reduced brain edema, prevented increased BBB permeability, reduced ICP and improved neurological scores. Second, ICI 182,780 eliminated the neuroprotective effects of estrogen mentioned above, showing that both ERα and ERβ may affect the neuroprotective effects of estrogen. Third, ICI 182,780 did not have relative agonistic effects for estrogen.

Our results show that the post-TBI administration of E2 in OVX rats causes a decrease in brain edema, permeability in BBB and ICP, and also improvement in neurologic scores in comparison to the vehicle group. The BBB damage after brain injury may result in gathering liquid in the extracellular space and consequently increasing the ICP [2]. On the other hand, the increase in ICP leads to the aggravation of neurologic state. It has been revealed that estrogen reduces BBB damage in the ischemia model through constraining the expression of the matrix metaloproteinase 2 and 9 genes and the vascular endothelial growth factor [19]. The positive effect of estrogen on the reduction of brain edema is probably due to its scavenger effect [20]. Other probable mechanisms of the neuroprotective effects of estrogen are the change of different gene expressions [21], the increase in brain perfusion [22], and the reduction in the activity of astrocytes and microglia [23]. The findings of the present study are in agreement with the work of Zhang *et al.* [24], who have found that the estrogen is able to reduce the volume of the damaged area and the cell death in brain ischemia. It has also been reported that estrogen causes the reduction of edema and the improvement in neurological scores following TBI [25]. Our previous studies showed that the administration of estrogen causes the reduction in brain edema [5] and the reduction of ICP [5, 7] following TBI in female rats. However, in another study no neuroprotective effects of estrogen have been found [26]. The reason for this difference in findings might be due to difference in the model and the intensity of induced injury, the drug dosage, and the period of drug usage.

In another part of this study, we indicated that prescribing ICI 182,780 along with estrogen eliminated the effects of estrogen such that the amount of brain edema, permeability of BBB, and ICP in the TBI + ICI + E2 group was not different from the TBI group, and no increase in the neurologic scores was observed after the use of estrogen. In spite of the significant difference between the TBI + E2 and the TBI + ICI + E2 groups, the neuroprotective effects of estrogen have not been completely eliminated. Therefore, there is the possibility that at least parts of the protective effects of estrogen are not dependent on classic estrogen receptors, or independent from estrogen receptors. The independent effects might be due to the intrinsic antioxidant attributes or might be mediated by nitric oxide [27]. It has been reported that ICI 182,780 functions as a pure antagonist of estrogen, competitively bonds with both nuclear and membrane (α and β) estrogen receptors and eliminates the effects of estrogen. This compound probably functions through one of the following mechanisms: increasing the degradation of ER [28], competing with estrogen receptor ligand for binding, restricting ER nuclei dimerization and localization, down-regulation of these two receptors, the restriction of transcriptions mediated by estrogen receptors [29], and reduction of available receptors. Until now, the restrictive effects of ICI 182,780 on estrogen protective effects after brain trauma have not been shown, and this is the first study that reports the effects of this compound on diffuse TBI. However, the restrictive effects of ICI 182,780 on estrogen in other brain injury models and also in other tissues have been shown. For instance, in MCAO, it has been indicated that ICI 182,780 restricts the effect of estrogen on reducing the volume of the infarcted area [30]. It restricts the effects of estrogen on the reduction of apoptosis in the brain cortex neurons [31]. In the trauma-hemorrahage, it eliminates the reductive effects of E2 on Ang II, AT1R, and myeloperoxidase activity [32]. There is also a study showing that the neuroprotective effects of estrogen are not eliminated by this compound (ICI) [33]. Possible reasons for such differences may be the differences in the studied injury model, studied tissue, and also used dose.

Considering the reports on agonistic effects of ICI 182,780 [24], we examined these effects in this stud. The results showed that when ICI 182,780 was used instead of estrogen 30 minutes after TBI, this compound did not have agnostic effects, meaning that it did not have neuroprotective effects similar to estrogen after TBI, when used alone. This result is supported by other studies as well [34, 35].

In this investigation, the neuroprotective mechanisms of estrogen were examined, and it was revealed that ERα and ERβ were effective in the neuroprotective function of estrogen. Nevertheless, there is a need for further studies to reveal the signalling pathways involved in the neuroprotective function of estrogen.

## References

[1] Mota BC, Pereira L, Souza MA, Silva LFA, Magni DV, Ferreira APO (2012). Exercise pre-conditioning reduces brain inflammation and protects against toxicity induced by traumatic brain injury: behavioral and neurochemical approach. Neurotox Res.

[2] Shlosberg D, Benifla M, Kaufer D, Friedman A (2010). Blood-brain barrier breakdown as a therapeutic target in traumatic brain injury. Nat Rev Neurol.

[3] Urban RJ (2006). Hypopituitarism after acute brain injury. Growth Horm IGF Res.

[4] Dang J, Mitkari B, Kipp M, Beyer C (2011). Gonadal steroids prevent cell damage and stimulate behavioral recovery after transient middle cerebral artery occlusion in male and female rats. Brain Behav Immun.

[5] Shahrokhi N, Khaksari M, Soltani Z, Mahmoodi M, Nakhaee N (2010). Effect of sex steroid hormones on brain edema, intracranial pressure, and neurologic outcomes after traumatic brain injury. Can J Physiol Pharmacol.

[6] Jover T, Tanaka H, Calderone A, Oguro K, Bennett MV, Etgen AM (2002). Estrogen protects against global ischemia-induced neuronal death and prevents activation of apoptotic signaling cascades in the hippocampal CA1. J Neurosci.

[7] Maghool F, Khaksari M (2013). Differences in brain edema and intracranial pressure following traumatic brain injury across the estrous cycle: involvement of female sex steroid hormones. Brain Res.

[8] Dubal DB, Zhu H, Yu J, Rau SW, Shughrue PJ, Merchenthaler I (2001). Estrogen receptor α, not β, is a critical link in estradiol- mediated protection against brain injury. Proc Natl Acad Sci.

[9] Miller NR, Jover T, Cohen HW, Zukin RS, Etgen AM (2005). Estrogen can act via estrogen receptor α and β to protect hippocampal neurons against global ischemia-induced cell death. J Endocrinol.

[10] Wise PM (2006). Estrogen therapy: does it help or hurt the adult and aging brain? Insights derived from animal models. J Neurosci.

[11] Wade GN, Blaustein JD, Gray JM, Meredith JM (1993). ICI 182,780: a pure antiestrogen that affects behaviors and energy balance in rats without acting in the brain. Am J Physiol RegulIntegr Comp Physiol.

[12] Chen D, Washbrook E, Sarwar N, Bates GJ, Pace PE, Thirunuvakkarasu V (2002). Phosphorylation of human estrogen receptor α at serine 118 by two distinct signal transduction pathways revealed by phosphorylation-specific antisera. Oncogene.

[13] Long X, Nephew KP (2006). Fluvestrant (ICI 182,780)-dependent interacting proteins mediate immobilization and degradation of estrogen receptor-alpha. J Biol Chem.

[14] Sawada M, Alkayed NJ, Goto S, Crain BJ, Traystman RJ, Shaivitz A (2000). Estrogen receptor antagonist ICI182, 780 exacerbates ischemic injury in female mouse. J Cereb Blood Flow Metab.

[15] Davis AM, Mao J, Naz B, Kohl JA, Rosenfeld CS (2008). Comparative effects of estradiol, methyl-piperidino-pyrazole, raloxifene, and ICI 182 780 on gene expression in the murine uterus. J Mol Endocrinol.

[16] Crandall C, Palla S, Reboussin B, Hu P, Barrett-Connor E, Reuben D (2006). Cross-sectional association between markers of inflammation and serum sex steroid levels in the postmenopausal estrogen/progestin interventions trial. J Womens Health (Larchmt).

[17] O'Connor CA, Cernak I, Vink R (2005). Both estrogen and progesterone attenuate edema formation following diffuse traumatic brain injury in rats. Brain Res.

[18] Maramarou A, Foda MA, van den Brink W (1994). A new model of diffuse brain injury in rats. J Neurosurg.

[19] Chi O, Barsoum S, Wen Y, Liu X, Weiss H (2004). 17β-Estradiol prevents blood-brain barrier disruption induced by VEGF. Horm Metab Res.

[20] Hoffman GE, Merchenthaler I, Zup SL (2006). Neuroprotection by ovarian hormones in animal models of neurological disease. J Endocr.

[21] Behl C, Holsboer F (1999). The female sex hormone oestrogen as a neuroprotectant. Trends Pharmacol Sci.

[22] Farhat M, Lavigne M, Ramwell P (1996). The vascular protective effects of estrogen. FASEB J.

[23] Baker AE, Brautigam VM, Watters JJ (2004). Estrogen modulates microglial inflammatory mediator production via interactions with estrogen receptor β. Endocrinology.

[24] Zhang YQ, Shi J, Rajakumar G, Day AL, Simpkins JW (1998). Effects of gender and estradiol treatment on focal brain ischemia. Brain Res.

[25] Shang Y, Brown M (2002). Molecular determinants for the tissue specificity of SERMs. Science.

[26] Theodorsson A, Theodorsson E (2005). Estradiol increases brain lesions in the cortex and lateral striatum after transient occlusion of the middle cerebral artery in rats: No effect of ischemia on galanin in the stroke area but decreased levels in the hippocampus. Peptides.

[27] Sunday L, Osuna C, Krause DN, Duckles SP (2007). Age alters cerebrovascular inflammation and effects of estrogen. Am J Physiol Heart Circ Physiol.

[28] Wittmann BM, Sherk A, McDonnell DP (2007). Definition of functionally important mechanistic differences among selective estrogen receptor down-regulators. Cancer Res.

[29] Mosselman S, Polman J, Dijkema R (1996). ERBeta: identification and characterization of a novel human estrogen receptor. FEBS Lett.

[30] Chiappetta O, Gliozzi M, Siviglia E, Amantea D, Morrone LA, Berliocchi L (2007). Evidence to implicate early modulation of interleukin‐1β expression in the neuroprotection afforded by 17β‐Estradiol in male rats undergone transient middle cerebral artery occlusion. Int Rev Neurobiol.

[31] Linford NJ, Dorsa DM (2002). 17 β-Estradiol and the phytoestrogen genistein attenuate neuronal apoptosis induced by the endoplasmic reticulum calcium-ATPase inhibitor thapsigargin. Steroids.

[32] Chen J, Yang S, Hu S, Choudhry MA, Bland KI, Chaudry IH (2008). Estrogen prevents intestinal inflammation after trauma-hemorrhage via downregulation of angiotensin II and angiotensin II subtype I receptor. Am J Physiol Gastrointest Liver Physiol.

[33] Patkar S, Farr T, Cooper E, Dowell F, Carswell H (2011). Differential vasoactive effects of oestrogen, oestrogen receptor agonists and selective oestrogen receptor modulators in rat middle cerebral artery. Neurosci Res.

[34] Das A, Smith JA, Gibson C, Varma AK, Ray SK, Banik NL (2011). Estrogen receptor agonists and estrogen attenuate TNF-α- induced apoptosis in VSC4 1 motoneurons. J Endocrinol.

[35] Di Liberto V, Mäkelä J, Korhonen L, Olivieri M, Tselykh T, Mäkelä A (2012). Involvement of estrogen receptors in the resveratrol-mediated increase in dopamine transporter in human dopaminergic neurons and in striatum of female mice. Neuropharmacology.

